# Editorial for “Materials Chemistry” Section, in Journal *Molecules*

**DOI:** 10.3390/molecules25225341

**Published:** 2020-11-16

**Authors:** Giulio Malucelli

**Affiliations:** Department of Applied Science and Technology, Local INSTM Unit, Politecnico di Torino, Viale Teresa Michel 5, 15121 Alessandria, Italy; giulio.malucelli@polito.it; Tel.: +39-0131-229369

Dear colleagues and friends, it is a great pleasure to summarize the most significant successes achieved during 2019 in the “Materials Chemistry” Section (https://www.mdpi.com/journal/molecules/sections/materials_chemistry) in the journal *Molecules*.

The “Materials Chemistry” Section of *Molecules* strives to provide an open access platform for disseminating high-quality articles and reviews at the core of the chemistry of materials. In this respect, the “Materials Chemistry” Section invites studies on the synthesis and characterization of either inorganic or organic (polymeric) materials, the investigation of structure–property relationships in metal, ceramic and polymer systems, as well as the design, preparation, and characterization of micro- and nano-composites (including hybrid organic-inorganic structures) and their applications in advanced sectors.

Furthermore, the total number of downloads/views of manuscripts published in *Molecules* in 2019 was 4,746,115, which, for the total of 4619 articles published, implies approximately 1000 views per article, demonstrating a superb visibility among both researchers and the scientific community. Finally, in terms of publication time, *Molecules* is among the fastest in the field, with an average of 32 days from submission to first publication. The rapid publishing time, while maintaining a rigorous peer-review process, is undeniably a credit to the dedication and professionalism of the Editors and the Editorial Team of *Molecules*.

It is clear that (1) a favorable and steadily increasing impact factor (IF = 3.267 (2019); 5-Year Impact Factor: 3.589 (2019)), (2) high visibility in open access publishing, (3) rapid publication times, and (4) the high quality of the rigorous peer-review process are among the benefits of publishing in the “Materials Chemistry” Section of *Molecules*.

In addition, there are some additional benefits. (1) We encourage authors and readers to take advantage of Special Issues that focus on a specific area of materials chemistry (at present the “Materials Chemistry” Section is running 127 Special Issues) and enjoy record visibility and higher readership than regular issues. (2) Finally, in the era of modern publishing with on-the-go mobile access, the Editorial Team at *Molecules* ensures that the published research enjoys rapid dissemination through a variety of platforms to reach everyone and maximize access. This includes Twitter, @Molecules_MDPI, Facebook and LinkedIn with the launch of regular highlights of “Hot Papers” and “Editors’ Choice” manuscripts.

A selection of cutting-edge “Hot-Papers” published in the Materials Chemistry Section of *Molecules* is presented at the end of this Editorial [1,2,3,4,5,6,7,8,9,10].

Materials chemistry is experiencing rapid growth. With the improvements in design and synthesis of new materials systems, characterization and analytical techniques, development of new technologies and novel micro- and nano-composite systems, the prospective contribution of materials chemistry research to the overall growth of materials science and technology seems limitless. We hope that you will consider submitting your material chemistry papers to Molecules.

When submitting materials chemistry papers to Molecules, “Materials Chemistry Section” should be selected in the scroll-down submission menu.

“Hot Articles” in the Materials Chemistry Section of Molecules:

**1.**  **Flame Retardant Epoxy Composites on the Road of Innovation: An Analysis with Flame Retardancy Index for Future Development** by Elnaz Movahedifar, Henri Vahabi, Mohammad Reza Saeb and Sabu Thomas

*Molecules* 2019, 24(21), 3964; https://doi.org/10.3390/molecules24213964

**2.**  **Chitosan-Based Bio-Composite Modified with Thiocarbamate Moiety for Decontamination of Cations from the Aqueous Media** by Nisar Ali, Adnan Khan, Muhammad Bilal, Sumeet Malik, Syed Badshah and Hafiz M. N. Iqbal

*Molecules* 2020, 25(1), 226; https://doi.org/10.3390/molecules25010226

**3.**  **Characterization and Structure–Property Relationships of Organic–Inorganic Hybrid Composites Based on Aluminum–Magnesium Hydroxycarbonate and Azo Chromophore** by Anna Marzec, Bolesław Szadkowski, Jacek Rogowski, Waldemar Maniukiewicz and Marian Zaborski

*Molecules* 2019, *24*(5), 880; https://doi.org/10.3390/molecules24050880

**4.**  **Anti-Biofilm Property of Bioactive Upconversion Nanocomposites Containing Chlorin e6 against Periodontal Pathogens** by Tianshou Zhang, Di Ying, Manlin Qi, Xue Li, Li Fu, Xiaolin Sun, Lin Wang and Yanmin Zhou

*Molecules* 2019, *24*(15), 2692; https://doi.org/10.3390/molecules24152692

**5.**  **Mechanical and Tribological Properties of Polytetrafluoroethylene Composites with Carbon Fiber and Layered Silicate Fillers** by Andrey P. Vasilev, Tatyana S. Struchkova, Leonid A. Nikiforov, Aitalina A. Okhlopkova, Petr N. Grakovich, Ee Le Shim and Jin-Ho Cho

*Molecules* 2019, *24*(2), 224; https://doi.org/10.3390/molecules24020224

**6.**  **Epoxy–PCM Composites with Nanocarbons or Multidimensional Boron Nitride as Heat Flow Enhancers** by Richa Agrawal, Joshua Hanna, I. Emre Gunduz and Claudia C. Luhrs

*Molecules* 2019, *24*(10), 1883; https://doi.org/10.3390/molecules24101883

**7.**  **Microwave Assisted Sol-Gel Synthesis of Silica-Spider Silk Composites** by Abul Bashar Mohammad Giasuddin and David W. Britt

*Molecules 2019*, *24*(14), 2521; https://doi.org/10.3390/molecules24142521

**8.**  **From Dermal Patch to Implants—Applications of Biocomposites in Living Tissues** by Karolina Papera Valente, Alexandre Brolo and Afzal Suleman

*Molecules* 2020, *25*(3), 507; https://doi.org/10.3390/molecules25030507

**9.**  **Mapping the Mechanical Properties of Hierarchical Supercrystalline Ceramic-Organic Nanocomposites** by Büsra Bor, Lydia Heilmann, Berta Domènech, Michael Kampferbeck, Tobias Vossmeyer, Horst Weller, Gerold A. Schneider and Diletta Giuntini

*Molecules* 2020, *25*(20), 4790; https://doi.org/10.3390/molecules25204790

**10.**  **All-Cellulose Composites: A Review of Recent Studies on Structure, Properties and Applications** by Behnaz Baghaei and Mikael Skrifvars

*Molecules* 2020, *25*(12), 2836; https://doi.org/10.3390/molecules25122836

## References

[1] Movahedifar E., Vahabi H., Reza Saeb M., Thomas S. (2019). Flame Retardant Epoxy Composites on the Road of Innovation: An Analysis with Flame Retardancy Index for Future Development. Molecules.

[2] Ali N., Khan A., Bilal M., Malik S., Badshah S., Iqbal H.M.N. (2020). Chitosan-Based Bio-Composite Modified with Thiocarbamate Moiety for Decontamination of Cations from the Aqueous Media. Molecules.

[3] Marzec A., Szadkowski B., Rogowski J., Maniukiewicz W., Zaborski M. (2019). Characterization and Structure–Property Relationships of Organic–Inorganic Hybrid Composites Based on Aluminum–Magnesium Hydroxycarbonate and Azo Chromophore. Molecules.

[4] Zhang T., Ying D., Qi M., Li X., Fu L., Sun X., Wang L., Zhou Y. (2019). Anti-Biofilm Property of Bioactive Upconversion Nanocomposites Containing Chlorin e6 against Periodontal Pathogens. Molecules.

[5] Vasilev A.P., Struchkova T.S., Nikiforov L.A., Okhlopkova A.A., Grakovich P.N., Shim E.L., Cho J.H. (2019). Mechanical and Tribological Properties of Polytetrafluoroethylene Composites with Carbon Fiber and Layered Silicate Fillers. Molecules.

[6] Agrawal R., Hanna J., Gunduz I.E., Luhrs C.C. (2019). Epoxy–PCM Composites with Nanocarbons or Multidimensional Boron Nitride as Heat Flow Enhancers. Molecules.

[7] Giasuddin A.B.M., Britt D.W. (2019). Microwave Assisted Sol-Gel Synthesis of Silica-Spider Silk Composites. Molecules.

[8] Valente K.P., Brolo A., Suleman A. (2020). From Dermal Patch to Implants—Applications of Biocomposites in Living Tissues. Molecules.

[9] Bor B., Heilmann L., Domènech B., Kampferbeck M., Vossmeyer T., Weller H., Schneider G.A., Giuntini D. (2020). Mapping the Mechanical Properties of Hierarchical Supercrystalline Ceramic-Organic Nanocomposites. Molecules.

[10] Baghaei B., Skrifvars M. (2020). All-Cellulose Composites: A Review of Recent Studies on Structure, Properties and Applications. Molecules.

